# Editorial: Multifaceted Approaches Combining Low or High LET Radiation and Pharmacological Interventions in Cancer and Radioprotection: From Bench to Bedside

**DOI:** 10.3389/fonc.2022.880607

**Published:** 2022-03-28

**Authors:** Pankaj Chaudhary, Sandeep Kumar Shukla, Shubhankar Suman

**Affiliations:** ^1^ The Patrick G Johnston Centre for Cancer Research, Faculty of Medicine, Health and Life Sciences, Queen’s University, Belfast, United Kingdom; ^2^ Institute of Nuclear Medicine & Allied Sciences, Defense Research and Development Organization (DRDO), Delhi, India; ^3^ Department of Oncology, Lombardi Comprehensive Cancer Center, Georgetown University Medical Center, Washington, DC, United States

**Keywords:** radiotherapy, cancer, radioprotection, radiosensitization, heavy ion radiotherapy, radiobiology

Radiotherapy (RT) using external or internal sources of ionizing radiation (IR) is a highly effective treatment modality to treat various cancer types (1, 2). Currently about half of all cancer patients receive RT during their lifetime often resulting in an overall increase in survival (3, 4). External beam radiotherapy (EBRT) mainly treat tumors employing photon (X-rays) or ion beams (proton and heavy ions) to the tumor tissue and are usually generated by a linear accelerator or cyclotron respectively, while brachytherapy (internal RT) uses internally implanted IR sources such as seeds, or capsules inside or in the close vicinity of the tumor tissue. Photon and electron beams based treatment modalities are still the most widely used form of EBRT, however, due to the high linear energy transfer (LET) characteristics protons and heavy ion radiation [such as Carbon (^12^C) ion] have been demonstrated to deposit relatively higher IR-dose specifically to the tumor region. Therefore, EBRT using protons and heavy-ions allows achieving greater normal tissue sparing, relative to photon beams (Malouff et al.) (5). While the technological developments related to treatment planning, image-guided beam delivery, bio-dosimetry to improve photon, proton, and heavy-ion EBRT is ongoing, identification of targetable IR-induced signaling pathways and development of pharmacological radio-modifiers (radiosensitizer and radioprotectors) are also required for further advancement of RT (5, 6). This special interdisciplinary issue presents research and review papers that touch upon biophysical, biological, and clinical aspects of RT.

RT-associated side effects are often associated with errors in bio-dosimetry, dose distribution patterns and individual sensitivity to develop acute and late tissue toxicity. Scattered particles and secondary neutrons may affect bio-dosimetry resulting in unexpected side effects after proton radiotherapy (PRT) and ^12^C-ion radiotherapy (7). The study by Horendeck et al.  confirmed that the Bragg peak and slightly shorter range of the post-Bragg peak region of proton radiation contain relatively high LET. This finding may explain the unwanted side effects, that could be improved using a narrower spread-out Bragg peak (SOBP). Additionally, the report by Buglewicz et al.  demonstrates that DNA double-strand breaks (DSBs) density increases with a decrease in cell survival at the Bragg peak. Further, they demonstrate differential changes in the DSB in the post-Bragg peak tail regions, which has implications in ^12^C-ion radiotherapy (CIRT) treatment planning to limit its late normal tissue toxicity and risk of secondary cancers.

RT with adjuvant chemotherapeutic agents is an established and classical approach for cancer therapy, however, an optimal combination of RT and targeted molecular therapy (including immunotherapy) is still not widely used (8, 9). A case report by Zhang suggests that a combination of RT and tyrosine kinase inhibitors (TKI) might be optimal in patients with brain metastasis of non-small-cell lung cancer (NSCLC) after resistance to crizotinib. Therefore, a detailed understanding of differential molecular responses in tumor and normal tissue after IR and molecular therapy is important for plausible target identification. The study by Macaeva et. al. demonstrated variable amplitude and timing of transcription response after photon and particle irradiation. A similar p53 related transcriptome was observed after photon and particle irradiation, however, immune response associated gene sets were significantly up-regulated in response to heavy ions. This indicates a higher immunogenic response of heavy ions and therefore optimization of PRT and immunotherapy combination might be required for better therapeutic outcomes. A review paper by Elbanna et. al. provides the perspective on clinical and preclinical aspects of combined RT and molecular therapy, however, most have failed primarily due to the lack of robust preclinical data. Additionally, timing and selection of molecular therapy with IR could also affect the outcome. While IR in the combination with immunotherapy could offer better control of the metastatic disease, and developing these combinations are often challenging due to discrepancies in the *in vitro*, *in vivo*, and clinical findings. The study by Reppigen et al.  emphasizes such discrepancies between *in vitro* and *in vivo* studies, where despite promising *in vitro* data, no synergistic effect of cabozantinib and IR was evident.

Despite the higher relative biological effectiveness (RBE) of PRT and CIRT for tumor cell killing, radiosensitizers are required to enhance their clinical efficacy (6). The study by Johnson et al.  tested the radiosensitization effect of hydroxamate-based histone deacetylase inhibitors (HDACi) and found that unlike radiosensitization observed with photon radiation, no appreciable tumor cell radiosensitization was achieved after proton and heavy-ions. Therefore, generalization of the widely used radiosensitizers with photons may not be appropriate for proton and heavy-ion therapy. In pursuit of the development of heavy-ion specific radiosensitizer, Prabhakaran et. al. studied the effects of ^12^C-ion in combination with fused toes homolog (FTS) silencing on uterine cervical cancer cells. The ^12^C-ion-induced overexpression of Notch signaling molecules was attenuated after silencing of FTS leading to an enhanced radiosensitization of the cervical cancer cells.

The recurrence of radioresistant tumors is also believed as one of the major factors restricting the efficacy and success of RT and some tumors respond poorly to conventional treatments after developing radioresistant phenotype (10). While several mechanisms implicated in the development of tumor radioresistance have been identified, the variation among tumor cell types is still a concern. In this direction, Yang et. al. have demonstrated that cancer-IgG regulated PI3K/AKT/DNA-PKcs signaling is associated with the development of radioresistance and poor prognosis of the lung. Using the siRNA screening approach, Nickson et. al. identified ubiquitin-specific protease 9X (USP9X) is required to stabilize key proteins involved in centrosome formation particularly in response to high-LET protons. They further demonstrated that the depletion of the (USP9X) in cancer cells (HeLa and UMSCC74A cells) using small interfering RNA (siRNA), led to a significant increase in the cell killing after high-LET radiation which can potentially increase the efficacy of protons and other high LET ions in treatment of radioresistant tumors.

Acute and late radiation toxicity is a typical adverse reaction in patients treated with RT or RT combined with chemotherapy (11), and there are no FDA-approved therapies to prevent or mitigate acute and late radiation toxicity after RT. The paper by García et. al. demonstrates that administration of recombinant Wnt5a before irradiation could confer radioprotection to normal gastrointestinal tissues. Additionally, using IR exposed mice, Sanguri and Gupta demonstrated gastrointestinal and hematopoietic tissue radioprotection by prebiotic mannan oligosaccharide pretreatment. Furthermore, using a 5-year phase-2 clinical survival study, Zhu et al., demonstrated epigallocatechin-3-Gallate as a potential treatment to alleviate esophagitis symptoms in small cell lung cancer patients exposed to IR without reducing survival.

Cancer is expected to continue threatening human life, as the number of new cancer diagnoses per year is expected to rise and RT is expected to be indispensable for cancer treatment. While EBRT is the mainstay for cancer RT, brachytherapy is still used to treat a subset of breast, prostate, cervix, and head and neck cancers. A review paper by Seniwal et al. provided an account of recent developments associated with the nanotechnology-based approach to improving brachytherapy. This paper also discusses the dosimetry protocols for nano-brachytherapy applications using radiolabeled nanoparticles (radio-NPs).

Finally, we appreciate the diverse participation from all the authors who responded to our call for this interdisciplinary issue and also extend our thanks to the esteemed reviewers who invested their valuable time to review the submitted manuscripts. We hope that this Research Topic will serve its intended purpose to give visibility and insight through important findings and ideas for the improvement of RT. We also hope that this Research Topic will gather attention from both basic radiobiologists and radiation oncologists.

## Author Contributions

PC, SKS, and SS equally contributed to writing, revising, and finalizing this editorial. All authors contributed to the article and approved the submitted version.

## Funding

PC acknowledges funding support from the Department of Economy, Northern Ireland, and Engineering and Physical Sciences Research Consortium (EPSRC) grant# EP/PO10059/1. SS acknowledges funding support from the National Aeronautics and Space Administration (NASA) grants # NNX09AU95G and #80NSSC18K1649.

## Conflict of Interest

The authors declare that the research was conducted in the absence of any commercial or financial relationships that could be construed as a potential conflict of interest.

## Publisher’s Note

All claims expressed in this article are solely those of the authors and do not necessarily represent those of their affiliated organizations, or those of the publisher, the editors and the reviewers. Any product that may be evaluated in this article, or claim that may be made by its manufacturer, is not guaranteed or endorsed by the publisher.
